# Associação entre insegurança alimentar e desenvolvimento infantil aos 18 meses do lactente na zona urbana de Pelotas, Rio Grande do Sul, Brasil

**DOI:** 10.1590/0102-311XPT198023

**Published:** 2025-02-07

**Authors:** Caroline Nickel Ávila, Jéssica Puchalski Trettim, Bárbara Borges Rubin, Carolina Coelho Scholl, Fernanda Teixeira Coelho, Mariana Bonati de Matos, Janaína Vieira dos Santos Motta, Ricardo Tavares Pinheiro, Luciana de Avila Quevedo

**Affiliations:** 1 Universidade Católica de Pelotas, Pelotas, Brasil.; 2 Universidade Federal de Pelotas, Pelotas, Brasil.

**Keywords:** Desenvolvimento Infantil, Segurança Alimentar e Nutricional, Insegurança Alimentar, Saúde Materno-Infantil, Child Development, Food and Nutrition Security, Food Insecurity, Maternal and Child Health, Desarrollo Infantil, Seguridad Alimentaria y Nutricional, Inseguridad Alimentaria, Salud Materno-Infantil

## Abstract

O objetivo deste estudo foi avaliar a associação entre a insegurança alimentar domiciliar e o desenvolvimento infantil aos 18 meses na cidade de Pelotas, Rio Grande do Sul, Brasil. Realizou-se estudo longitudinal com uma amostra de base populacional de 465 mães e lactentes. Os desenvolvimentos cognitivo, motor, socioemocional e de linguagem dos lactentes foram avaliados através da terceira edição da *Escala Bayley do Desenvolvimento do Bebê e da Criança Pequena*. A insegurança alimentar foi mensurada por meio da *Escala Brasileira de Insegurança Alimentar* que classifica os domicílios em segurança alimentar ou insegurança alimentar leve, moderada ou grave. Os resultados mostraram que, após análise ajustada, apenas os desenvolvimentos motor e socioemocional sofreram efeito da insegurança alimentar aos 18 meses. A cada aumento do nível de insegurança alimentar, o escore de desenvolvimento motor diminuiu, em média, 2,30 pontos (IC95%: -4,31; -0,48) aos 18 meses de idade. Similarmente, a cada aumento do nível de insegurança alimentar, o escore de desenvolvimento socioemocional decresceu, em média, 4,05 pontos (IC95%: -7,34; -0,76). Os resultados evidenciam, portanto, que a insegurança alimentar foi associada a menores desenvolvimentos motor e socioemocional aos 18 meses do lactente, enfatizando a importância do direito à alimentação adequada e da existência de ambientes que forneçam experiências estimulantes para o desenvolvimento infantil.

## Abstract (en)

This study aimed to evaluate the association between household food insecurity and child development at age 18 months in the municipality of Pelotas, Rio Grande do Sul State, Brazil. This longitudinal study was carried out with a population-based sample of 465 mothers and infants from Pelotas. Infants’ cognitive, motor, socioemotional, and language developments were assessed by the *Bayley Scales of Infant and Toddler Development*, third edition. The *Brazilian Food Insecurity Scale* was used to define food safety/insecurity, which classifies households into four categories: food security or mild, moderate, or severe food insecurity. The results of the adjusted analysis showed that food insecurity only affected motor and socioemotional developments at age 18 months. Each increase in food insecurity score decreased that of motor development by 2.30 points on average (95%CI: -4.31; -0.48) at age 18 months. Similarly, each increase in food insecurity score decreased that of socioemotional development by 4.05 points on average (95%CI: -7.34; -0.76). Results show that food insecurity was associated with lower motor and socioemotional developments at age 18 months and stress the importance of the right to adequate food and to environments that provide stimulating experiences for child development.

## Resumen

El objetivo de este estudio fue evaluar la asociación entre la inseguridad alimentaria en el hogar y el desarrollo infantil a los 18 meses en la ciudad de Pelotas, Rio Grande do Sul, Brasil. Este es un estudio longitudinal con una muestra poblacional de 465 madres y lactantes de la ciudad de Pelotas. Los desarrollos cognitivo, motor, socioemocional y del lenguaje de los lactantes se evaluaron mediante la tercera edición de la *Escala Bayley de Desarrollo de Bebés y Niños*. La inseguridad alimentaria se midió utilizando la *Escala Brasileña de Inseguridad Alimentaria*, que clasifica a los hogares en seguridad alimentaria o inseguridad alimentaria (leve, moderada o grave). Los resultados mostraron que, después de un análisis ajustado, solo los desarrollos motor y socioemocional sufrieron el efecto de la inseguridad alimentaria a los 18 meses. Cada aumento en el nivel de inseguridad alimentaria llevó a una disminución en la puntuación de desarrollo motor en un promedio de 2,30 puntos (IC95%: -4,31; -0,48) a los 18 meses de edad. Del mismo modo, cada aumento en el nivel de inseguridad alimentaria llevó a una disminución en la puntuación de desarrollo socioemocional en un promedio de 4,05 puntos (IC95%: -7,34; -0,76). Los resultados muestran que la inseguridad alimentaria se asoció con menores desarrollos motor y socioemocional a los 18 meses del lactante, lo que indica la importancia del derecho a una alimentación adecuada y la existencia de entornos que brinden experiencias estimulantes para el desarrollo infantil.

## Introdução

A insegurança alimentar é uma condição caracterizada por preocupação e angústia, por parte dos moradores do domicílio, diante da incerteza de dispor regularmente de alimentos nutricionalmente adequados e seguros para o consumo. Essa condição pode ser classificada em três níveis: leve, moderada e grave. Considera-se insegurança alimentar leve quando há preocupação quanto à adequação do abastecimento alimentar doméstico, havendo redução da qualidade dos alimentos, aumento dos padrões de adaptação alimentar e pouca ou nenhuma redução na ingestão de alimentos. O nível moderado caracteriza-se pela redução da ingestão alimentar, principalmente entre os adultos, podendo atingir também as crianças em algumas famílias. Já o nível grave é definido pela redução do consumo alimentar por todos os membros da família, inclusive as crianças. Nessa última, pode decorrer uma subalimentação devido à real escassez de alimentos ^1^ ^,^ ^2^.

Considerada um problema persistente no cenário brasileiro, a insegurança alimentar voltou a aumentar a partir de 2015 após anos de diminuição, quando o país saiu do Mapa da Fome da Organização das Nações Unidas (ONU) ao apresentar menos de 5% da população em situação de subalimentação ^3^. Esse aumento nos últimos anos pode ser atribuído à crise político-econômica iniciada em 2015, aos desmontes das políticas de segurança alimentar e nutricional, e à pandemia que se instalou em 2020, agravando ainda mais a situação de insegurança alimentar e suas consequências para a população ^4^.

Segundo a *Pesquisa de Orçamentos Familiares* (POF), realizada entre os anos de 2017 e 2018, revelou-se que 36,7% dos domicílios - o equivalente a 25,3 milhões - apresentavam algum grau de insegurança alimentar ^5^. Mais recentemente, o segundo *Inquérito Nacional sobre Insegurança Alimentar no Contexto da Pandemia da COVID-19 no Brasil* ^6^ mostrou que mais da metade da população brasileira vivia nessa condição, sendo que 30,7% compreendiam os níveis moderado a grave. Ainda, quase um terço dos domicílios brasileiros com crianças menores de cinco anos de idade está nessa condição ^7^ ^,^ ^8^.

A insegurança alimentar pode causar prejuízos no desenvolvimento infantil por meio de vários mecanismos. Atrasos no desenvolvimento global e na dimensão socioemocional são observados na literatura ^9^. Crianças nessa condição podem se tornar menos ativas, mais distraídas e irritadas, reduzindo o nível de interações estimulantes com seus cuidadores e comprometendo seu desenvolvimento ^10^ ^,^ ^11^. Além disso, uma situação persistente de insegurança alimentar pode resultar em níveis mais baixos de diferentes habilidades cognitivas e emocionais. Entretanto, prejuízos já são observados uma vez que a criança tenha vivenciado a condição ^12^.

As crianças com insegurança alimentar são mais propensas a ter dietas de baixa qualidade que podem levar a deficiências de vitaminas e minerais essenciais. Tais deficiências caracterizam o que chamamos de “fome oculta”, isto é, o indivíduo tem acesso a alimentos com quantidade suficiente de calorias, mas escassos em micronutrientes essenciais, como vitaminas e minerais. A fome oculta não se manifesta de forma visível, como os sintomas da fome clássica, dificultando sua detecção precoce e podendo levar a comprometimentos significativos no funcionamento cerebral e nas capacidades cognitivas e motoras a longo prazo ^13^ ^,^ ^14^ ^,^ ^15^. A literatura tem demonstrado que o consumo calórico adequado e o gasto energético basal estão diretamente associados ao desempenho cognitivo e motor, sendo fundamentais para a manutenção das funções cerebrais e para a plasticidade neuronal ^16^ ^,^ ^17^.

O desenvolvimento infantil é um processo que se inicia na vida intrauterina e envolve aspectos como o crescimento físico, a maturação neurológica e as aquisições de habilidades relacionadas ao comportamento e às esferas cognitiva, motora, afetiva e social. O período compreendido pelos primeiros mil dias de vida de um lactente - 270 dias referentes à gestação e 730 dias relacionados aos primeiros 24 meses de idade - é consagrado como uma fase primordial para o desenvolvimento e o crescimento infantil em níveis adequados ^18^. Esse período constitui uma janela de oportunidades que podem impactar na qualidade de vida do lactente, em prejuízos no estado de saúde/doença ao longo da vida e no desenvolvimento infantil em curto prazo ^19^. A partir disso, os primeiros anos de vida são marcados pela importante formação e aceleração do desenvolvimento dessas habilidades ^20^, que visam torná-lo competente para responder às suas necessidades e às necessidades do meio em que vive ^21^.

Nesse contexto, a literatura evidencia diversos estudos que avaliam as habilidades acadêmicas de crianças e adolescentes em fases pré-escolar e escolar, não considerando os primeiros mil dias de vida e nem a gravidade da insegurança alimentar. Além disso, a maioria dos estudos foram realizados em países com contextos socioculturais diferentes do Brasil ^22^ ^,^ ^23^. Dessa forma, considerando a importância dos primeiros mil dias de vida para o crescimento e o desenvolvimento do lactente, este estudo pretende preencher essa lacuna, avaliando a associação entre a presença de insegurança alimentar domiciliar e o desenvolvimento infantil do lactente aos 18 meses na cidade de Pelotas, Estado do Rio Grande do Sul.

## Métodos

Trata-se de um estudo longitudinal aninhado a um estudo de coorte intitulado *Transtornos Neuropsiquiátricos Maternos no Ciclo Gravídico-Puerperal: Detecção e Intervenção Precoce e suas Consequências na Tríade Familiar*, composto por sete fases de avaliação ^24^.

O processo de amostragem foi realizado em múltiplos estágios, tendo sido selecionados, de forma sistemática, 50% dos setores censitários da zona urbana de Pelotas, delimitados pelo Instituto Brasileiro de Geografia e Estatística (IBGE). Primeiro, foram listados os 488 setores censitários da zona urbana, de acordo com a malha do *Censo Demográfico* de 2010, para posterior sorteio de 244 setores. Entre os anos de 2016 e 2018, cada setor sorteado recebeu a visita de um entrevistador treinado para a listagem de todos os domicílios com gestantes de até 24 semanas. Foram identificadas 1.073 gestantes nos primeiros dois trimestres de gravidez, no qual 983 participaram da primeira avaliação. Entre os anos de 2018 e 2020, aos 18 meses do lactente, todas as mães e seus filhos que participaram da primeira fase de avaliação foram contatadas e convidadas a participar da quarta fase. Mulheres com deficiência visual e aquelas que apresentavam alguma incapacidade de compreender e/ou responder o questionário foram excluídas da amostra.

A amostra deste estudo foi composta por 465 mães e seus respectivos filhos que participaram da quarta fase de avaliação (18 meses pós-parto) e que eram residentes na zona urbana da cidade de Pelotas. Esse município fica no extremo sul do Brasil, o qual tem uma população estimada de 325.685 habitantes, sendo o quarto mais populoso do estado ^25^. De acordo com o último *Censo Demográfico*, Pelotas contém mais de 6.700 crianças com idades entre 1 e 2 anos residindo nas zonas urbana e rural ^25^. A partir disso, nossa amostra representa 6,9% dessas crianças.

Para avaliar o desenvolvimento dos lactentes aos 18 meses de vida foi utilizada a terceira edição da *Escala Bayley do Desenvolvimento do Bebê e da Criança Pequena* (*Bayley Scales of Infant and Toddler Development, Third Edition* - Bayley-III), em sua versão traduzida para o Brasil ^26^. Trata-se de uma escala administrada individualmente que avalia os cinco principais domínios de desenvolvimento em crianças entre 16 dias e 42 meses de idade. É dividida em: cognição, linguagem (receptiva e expressiva), motricidade (grossa e fina), socioemocional e comportamento adaptativo. Os três primeiros domínios são avaliados por meio da observação durante a aplicação dos itens com a criança, e os dois últimos são avaliados por meio de questionários respondidos pelos pais ou cuidadores. Para este estudo, foram utilizados os domínios cognitivo, motor, de linguagem e socioemocional. Todas as escalas foram administradas por profissionais da saúde devidamente treinados na aplicação do instrumento. Cada dimensão apresenta um escore bruto que é transformado em escore composto, de acordo com a idade da criança e ajustado para prematuridade. A partir dos escores compostos, é possível determinar uma classificação em percentil. Entretanto, uma vez que as tabelas normativas são baseadas em uma amostra norte-americana e considerando que tal classificação não tem validação para a população brasileira, para este estudo, foi utilizado o escore composto tratado como contínuo para a realização das análises. Os escores compostos das escalas variam de 40 a 160 pontos. Para a interpretação dos resultados, quanto maior o escore, melhor o desenvolvimento infantil.

Para mensurar a insegurança alimentar, foi utilizada a *Escala Brasileira de Insegurança Alimentar* (EBIA). Ela é composta por 14 itens sobre a experiência vivenciada pela família com relação à suficiência alimentar nos últimos três meses que antecederam a entrevista, sendo oito destinados a famílias sem indivíduos menores de 18 anos e um adicional de seis itens para famílias que contêm pelo menos um indivíduo menor de 18 anos residente no domicílio. As perguntas têm duas alternativas (não e sim), em que cada resposta afirmativa representa um ponto. A pontuação final da escala é resultante da somatória dos pontos, sendo classificada em segurança alimentar (0 pontos), insegurança alimentar leve (1-5 pontos em famílias com menores de 18 anos ou 1-3 em famílias sem indivíduos menores), insegurança alimentar moderada (6-10 pontos nas famílias com menores de 18 anos ou 4-6 nas famílias sem indivíduos menores) e insegurança alimentar grave (11-14 pontos nas famílias com menores de 18 anos ou 7-8 nas famílias sem indivíduos menores) ^1^ ^,^ ^2^. Para fim das análises deste estudo, as categorias de insegurança alimentar moderada e grave foram agrupadas em uma só categoria.

As seguintes variáveis independentes foram consideradas no presente estudo para fins de controle de confundidores: idade materna em anos (≤ 26, 27-32, ≥ 33); escolaridade materna em anos (≤ 8, ≥ 9); sexo do lactente (masculino, feminino); cor da pele do lactente (branca, preta, parda); prematuridade < 37 semanas (não, sim); baixo peso ao nascer < 2.500g (não, sim); intercorrências no nascimento (não, sim); lactente frequenta creche (não, sim); pai biológico vive no domicílio (não, sim); tempo de amamentação em meses (menor que 12, 12 ou mais) e insegurança alimentar na gestação (segurança alimentar, insegurança alimentar leve, insegurança alimentar moderada e grave).

As análises estatísticas foram realizadas no programa IBM SPSS 26.0 (https://www.ibm.com/). Para a análise descritiva foram apresentadas as frequências absolutas e relativas. Nas análises bivariadas, foi utilizado análise de variância (ANOVA) com *post hoc* de Bonferroni para comparar as médias dos escores de desenvolvimento infantil e as diferentes categorias de insegurança alimentar. Os pressupostos da ANOVA foram testados pelo teste de Levene que revelou homocedasticidade dos dados (p > 0,05). No ajuste de possíveis fatores de confusão, foi utilizada a regressão linear com cinco níveis hierárquicos de ajuste (nível 1 - características maternas; nível 2 - características do nascimento do lactente; nível 3 - variáveis lactente frequenta creche e pai biológico vive no domicílio; nível 4 - tempo de amamentação; e nível 5 - insegurança alimentar na gestação). Em todas as análises, assumiu-se um nível de significância de 5% e um poder de 80%.

A pesquisa foi aprovada pelo Comitê de Ética em Pesquisa da Universidade Católica de Pelotas (UCPel; parecer nº 47807915.4.0000.5339) e todas as mães consentiram a sua participação e a do lactente através da assinatura do Termo de Consentimento Livre e Esclarecido (TCLE). Todos os participantes identificados com insegurança alimentar (moderada e grave) foram encaminhados ao Centro de Referência em Assistência Social (CRAS) mais próximo de sua residência e todos os lactentes identificados com baixo escore de desenvolvimento infantil foram encaminhados para o ambulatório de pediatria da UCPel.

## Resultados

A amostra foi composta por 465 mães e seus filhos aos 18 meses pós-parto. A maioria das mulheres tinha entre 27 e 32 anos de idade (34,6%) e nove anos ou mais de escolaridade (75%). Com relação aos lactentes, a maioria era do sexo feminino (51,6%) e de cor da pele branca (72,5%), 54 (13,4%) nasceram prematuros, 38 (9,4%) nasceram com baixo peso e 47 (11,5%) tiveram algum tipo de intercorrência no nascimento. A maioria dos lactentes não frequentava creche (66,9%), vivia com o pai biológico no domicílio (81,7%) e foi amamentado por 12 meses ou mais (61,1%) (Tabela 1).

**Tabela 1:** Caracterização da amostra segundo as variáveis de características de mães e lactentes aos 18 meses de idade na cidade de Pelotas, Rio Grande do Sul, Brasil, 2016-2020.

Variáveis	n	%
Idade materna (anos)		
≤ 26	153	32,9
27-32	161	34,6
≥ 33	151	32,5
Escolaridade materna (anos) *		
≤ 8	116	25,0
≥ 9	348	75,0
Sexo do lactente		
Masculino	225	48,4
Feminino	240	51,6
Cor da pele do lactente *		
Branca	334	72,5
Preta	59	12,8
Parda	68	14,8
Prematuridade (< 37 semanas) *		
Não	350	86,6
Sim	54	13,4
Baixo peso ao nascer (< 2.500g) *		
Não	368	90,6
Sim	38	9,4
Intercorrências no nascimento *		
Não	361	88,5
Sim	47	11,5
Lactente frequenta creche		
Não	311	66,9
Sim	154	33,1
Pai biológico vive no domicílio		
Não	85	18,3
Sim	380	81,7
Tempo de amamentação (meses) *		
Menor que 12 meses	181	38,9
12 meses ou mais	284	61,1
Insegurança alimentar na gestação		
Segurança alimentar	315	67,7
Insegurança alimentar leve	120	25,8
Insegurança alimentar moderada/grave	30	6,5
Insegurança alimentar aos 18 meses *		
Segurança alimentar	323	69,6
Insegurança alimentar leve	103	22,2
Insegurança alimentar moderada/grave	38	8,2
Total	465	100,0

* Variáveis com ausência de dados.

Quanto à segurança alimentar, 38 (8,2%) lactentes estavam em situação de insegurança alimentar moderada/grave aos 18 meses. A média do desenvolvimento cognitivo dos lactentes foi de 96,1 pontos (desvio padrão - DP: 14,1), do desenvolvimento motor foi de 99,4 pontos (DP: 11,0), da linguagem foi 95,7 pontos (DP: 15,4) e a do desenvolvimento socioemocional foi de 100,4 pontos (DP: 19,8).

**Figura 1:**
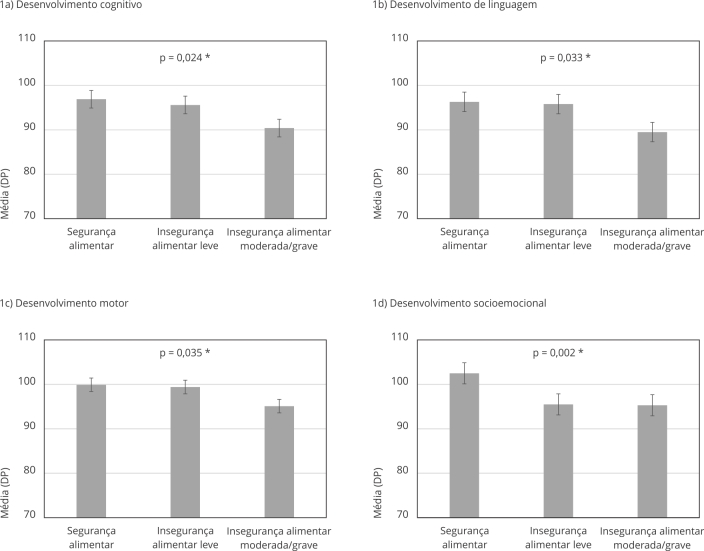
Médias do desenvolvimento infantil aos 18 meses de acordo com a presença de insegurança alimentar no mesmo período.

A Figura 1 apresenta as médias (M) de desenvolvimento infantil de acordo com as categorias de segurança alimentar. Com relação ao desenvolvimento cognitivo, lactentes com insegurança alimentar moderada/grave tiveram pior desempenho (M = 90,4; DP: 11,6) em comparação aos lactentes com segurança alimentar (M = 96,9; DP: 14,6; p = 0,021). Quanto ao desenvolvimento motor, lactentes com insegurança alimentar moderada/grave também tiveram pior desempenho (M = 95,1; DP: 11,5) do que lactentes com segurança alimentar (M = 99,9; DP: 10,6; p = 0,029). Da mesma forma, lactentes com insegurança alimentar moderada/grave tiveram pior desempenho no desenvolvimento da linguagem (M = 89,5; DP: 11,9) em comparação aos lactentes com segurança alimentar (M = 96,3; DP: 16,0; p = 0,028). Considerando o desenvolvimento socioemocional, lactentes com insegurança alimentar leve tiveram pior desempenho (M = 95,5; DP: 17,5) quando comparados aos lactentes com segurança alimentar (M = 102,5; DP: 19,7; p = 0,005).

* Análise de variância (ANOVA).

Após a análise ajustada, apenas os desenvolvimentos motor e socioemocional sofreram efeito da insegurança alimentar aos 18 meses. A cada aumento do nível de insegurança alimentar, o escore de desenvolvimento motor diminuiu, em média, 2,30 pontos (intervalo de 95% de confiança - IC95%: -4,13; -0,48) aos 18 meses de idade. Similarmente, a cada aumento do nível de insegurança alimentar, o escore de desenvolvimento socioemocional decresceu, em média, 4,05 pontos (IC95%: -7,34; -0,76) (Tabela 2).

**Tabela 2:** Regressão linear ajustada dos escores de desenvolvimento infantil de acordo com a insegurança alimentar de lactentes aos 18 meses de idade na cidade de Pelotas, Rio Grande do Sul, Brasil, 2016-2020.

Variável	Desenvolvimento infantil											
	Cognitivo			Motor			Linguagem			Socioemocional		
	B	IC95%	Valor de p	B	IC95%	Valor de p	B	IC95%	Valor de p	B	IC95%	Valor de p
Insegurança alimentar aos 18 meses (segurança alimentar *)	-1,94	-4,31; 0,44	0,109	-2,30	-4,13; -0,48	0,014	-1,88	-4,29; 0,53	0,126	-4,05	-7,34; -0,76	0,016

IC95%: intervalo de 95% de confiança.

Nota: controlado para as variáveis idade materna, escolaridade materna, sexo do lactente, cor da pele do lactente, prematuridade, baixo peso ao nascer, intercorrências no nascimento, lactente frequenta creche, pai biológico vive no domicílio, tempo de amamentação e insegurança alimentar na gestação.

* Categoria de referência.

## Discussão

Este estudo fornece dados sobre os desenvolvimentos cognitivo, motor, de linguagem e socioemocional de lactentes aos 18 meses de vida e sua relação com a condição de insegurança alimentar. Os resultados aqui apresentados sugerem que a insegurança alimentar pode produzir efeitos adversos sobre os desenvolvimentos motor e socioemocional dos lactentes nessa idade.

O contexto de insegurança alimentar tem papel importante no desenvolvimento infantil. A relação entre a presença de insegurança alimentar e a diminuição do escore do desenvolvimento motor dos lactentes foi evidenciada por este estudo. Tal achado é consistente com pesquisas que demonstram os efeitos negativos da insegurança alimentar e da fome oculta no desenvolvimento motor infantil ^15^ ^,^ ^27^. Milner et al. ^10^ revelaram que crianças com insegurança alimentar são mais propensas a apresentar atrasos no alcance de marcos motores importantes, como ficar em pé, caminhar e equilibrar-se. A falta de nutrientes essenciais prejudica o desenvolvimento adequado dos músculos e do sistema nervoso, cruciais para o desenvolvimento motor ^14^.

De forma similar, foi encontrada associação entre a insegurança alimentar e o desenvolvimento socioemocional do lactente. Esse achado é consistente com pesquisas recentes, incluindo uma revisão que evidencia uma relação direta entre a falta de acesso regular a alimentos nutritivos e o impacto adverso no bem-estar emocional e comportamental de crianças e adolescentes, sugerindo que a insegurança alimentar pode ter efeitos duradouros no desenvolvimento socioemocional, afetando a capacidade das crianças de formar relacionamentos saudáveis e a sua autoestima ^23^. Nesse sentido, a condição de insegurança alimentar está fortemente associada a desafios psicossociais ^28^, como o estresse familiar devido à condição de restrição alimentar, e pode levar à redução do nível de interações estimulantes dos lactentes com seus cuidadores, comprometendo seu desenvolvimento socioemocional ^10^ ^,^ ^11^. Esses resultados sublinham a importância de abordagens integradas que não apenas garantam o acesso adequado a alimentos, mas também promovam um ambiente emocionalmente seguro para o desenvolvimento saudável dos lactentes.

A insegurança alimentar influencia a saúde e o desenvolvimento por meio dos seus efeitos na nutrição e como componente do estresse familiar geral, contribuindo para um ambiente disfuncional físico e psicoemocional que representa um risco para o desenvolvimento ideal do lactente. É possível, portanto, que a associação entre insegurança alimentar e os desenvolvimentos motor e socioemocional dos lactentes aos 18 meses esteja relacionada ao tempo de exposição da família à condição de insegurança alimentar. Ou seja, é possível que a insegurança alimentar exponha efeitos mais imediatos nos desenvolvimentos motor e socioemocional em comparação aos desenvolvimentos cognitivo e de linguagem, que podem demandar mais tempo de exposição à insegurança alimentar para expor algum tipo de associação. Nessa perspectiva, a insegurança alimentar está associada ao desenvolvimento infantil não apenas por fatores nutricionais, mas também por questões psicoemocionais que afetam a unidade familiar como um todo ^29^.

Nos primeiros cinco anos de vida ocorrem mudanças marcantes nos hábitos alimentares e nos desenvolvimentos cognitivo, linguístico, social e emocional, caracterizando-se como de maior vulnerabilidade biológica. Por isso, é prioritário garantir a esse grupo etário o acesso regular e permanente à alimentação, pois experimentar restrição alimentar quantitativa importante, ou episódios que configuram situação de fome durante esse período, reflete negativamente no desenvolvimento neuropsicomotor dos futuros cidadãos do país ^30^.

Neste estudo, não foi encontrada associação entre insegurança alimentar e desenvolvimento cognitivo e de linguagem infantil. É importante ressaltar que a associação entre essas variáveis não é uma conclusão universal entre os estudos disponíveis na literatura ^22^ ^,^ ^23^. Estudos utilizam diferentes metodologias e escalas para medir tanto a insegurança alimentar quanto os desenvolvimentos cognitivo e de linguagem. Essa variação pode levar a resultados inconsistentes entre os estudos, dependendo de como as variáveis são definidas e medidas. Além disso, a gravidade e a duração da insegurança alimentar podem variar significativamente entre os estudos e dentro das populações estudadas. Isso pode influenciar a magnitude dos efeitos observados nos desenvolvimentos cognitivo e de linguagem dos lactentes, podendo ser mais sutis ou só se tornarem aparentes ao longo do tempo. Ainda, as populações estudadas variam em termos de composição demográfica, contexto socioeconômico e cultural. Essas diferenças podem influenciar como a insegurança alimentar afeta os desenvolvimentos cognitivo e de linguagem ^22^ ^,^ ^23^.

Este estudo apresenta algumas limitações; entre estas, destaca-se a perda de acompanhamento ocorrida devido à pandemia de COVID-19, resultando em uma amostra menor do que a estimada. Vale ressaltar ainda que, uma vez que a amostra deste estudo compreende apenas mães e seus filhos residentes na zona urbana da cidade de Pelotas, não há representatividade amostral da população geral do município. Além disso, apesar de ser de um estudo longitudinal com variáveis coletadas em outras fases do acompanhamento, a associação entre a insegurança alimentar e o desenvolvimento infantil é transversal, impossibilitando a relação temporal, uma vez que existe a possibilidade de causalidade reversa para as associações entre essas variáveis. E pode haver, ainda, viés de memória relacionado às questões sobre insegurança alimentar, visto que se referem aos últimos três meses que antecederam a entrevista. Por fim, não foram coletadas informações sobre aleitamento materno exclusivo.

Contudo, a realização deste estudo salienta aspectos positivos. Destaca-se o fato de ser um estudo de base populacional, que acompanha mães e filhos desde a gestação. Além disso, para avaliar o desenvolvimento infantil, foi utilizada a Bayley-III, em sua versão traduzida para o Brasil, garantindo a possibilidade de comparabilidade de aplicação em outros países. A avaliação do desenvolvimento dos lactentes foi realizada por profissionais de saúde, discentes de um programa de pós-graduação previamente treinados, e permitiu a identificação precoce de problemas e atrasos no desenvolvimento, possibilitando, após a avaliação, orientações de estímulos adequados a fim de evitar prejuízos futuros no desenvolvimento. Da mesma forma, a EBIA é considerada um método direto de medir a condição domiciliar de insegurança alimentar e foi proposta e validada para sua utilização no Brasil por Segall-Corrêa et al. ^2^ por meio da adaptação da escala do Departamento de Agricultura dos Estados Unidos ^1^, o que também garante a possibilidade em comparabilidade.

De maneira geral, os resultados deste estudo apontam importantes efeitos produzidos pela associação entre a insegurança alimentar e os desenvolvimentos motor e socioemocional dos lactentes aos 18 meses de idade. Abordar essa questão por meio de políticas públicas e intervenções específicas é crucial para garantir o desenvolvimento saudável e o bem-estar dos lactentes. Em vista disso, para a efetivação do adequado desenvolvimento infantil e da segurança alimentar e nutricional deve-se considerar o direito à alimentação de qualidade em quantidades suficientes por meio do aprimoramento de políticas públicas de alimentação e nutrição. Além disso, a realização do direito da educação infantil irá propiciar a existência de ambientes sociais que forneçam diferentes e enriquecedoras experiências estimulantes para que o desenvolvimento infantil adequado se torne uma realidade para todos os lactentes. Por fim, a realização de novos estudos é encorajada para melhor compreensão dos mecanismos pelos quais a insegurança alimentar afeta o desenvolvimento infantil e para identificar estratégias eficazes de mitigação.
